# 652. The Application of Human Reliability Analysis to Meek Micrografting in Major Burns Surgery

**DOI:** 10.1093/jbcr/irag033.489

**Published:** 2026-04-06

**Authors:** Patrick K O'Donohoe, Sharon Kennedy, Odhran Shelley, Laura Pompermaier

**Affiliations:** St James's Hospital, Dublin, Ireland; St James's Hospital, Dublin, Ireland; St James's Hospital, Dublin, Ireland; Linköping University, Sweden Centre for Disaster Medicine and Traumatology, Linköping, Sweden, Norrköping, Ostergotlands Lan

## Abstract

**Introduction:**

Meek micrografting is an invaluable tool in the management of major burns allowing for significant expansion of skin autograft. Several authors have previously noted its steep learning curve, which may discourage uptake. Human Reliability Analysis is a methodology that provides a structured approach to examining skills acquisition and human error, which we have applied to Meek micrografting.

**Methods:**

The process of Meek micrografting was broken down into individual steps and subtasks through review of the literature on the technique and by direct observation of experts, with a structured review of these through Hierarchical Task Analysis (HTA). The Systematic Human Error Reduction and Prediction Approach (SHERPA) was then applied to this process and areas of risk were identified by consensus of subject matter experts.

**Results:**

In total,17 papers describing Meek micrografting between 1993 and 2024 were identified, describing the technique as used in 300 patients. Through this literature review and direct observation of the technique, Meek micrografting was broken down into 8 steps with 37 subtasks required to successfully complete the procedure. Areas of potential error that were identified included those related to correct use of equipment, timing of critical steps, and appropriate post op care of the grafts. Potential solutions were suggested for each error.

**Conclusions:**

Human Reliability Analysis allows a complex task like Meek micrografting to be broken down into simple subtasks through which likely or serious errors can be identified and remedied. This may help improve training of surgical teams, reduce error, and improve patient safety.

**Applicability of Research to Practice:**

This Human Reliability Analysis can provide a framework for training and acquisition of skills in Meek micrografting, including in units where it has not previously been utilised.

**Funding for the study:**

N/A.

